# Electrical Transport and Thermoelectric Properties of SnSe–SnTe Solid Solution

**DOI:** 10.3390/ma12233854

**Published:** 2019-11-22

**Authors:** Jun-Young Cho, Muhammad Siyar, Woo Chan Jin, Euyheon Hwang, Seung-Hwan Bae, Seong-Hyeon Hong, Miyoung Kim, Chan Park

**Affiliations:** 1 Department of Materials Science and Engineering, Seoul National University, Seoul 08826, Korea; takecjy@gmail.com (J.-Y.C.); woottan@snu.ac.kr (W.C.J.); shhong@snu.ac.kr (S.-H.H.); mkim@snu.ac.kr (M.K.); 2 School of Chemical & Materials Engineering, National University of Sciences and Technology, Islamabad H–12, Pakistan; engrsiyar.uet@gmail.com; 3 SKKU Advanced Institute of Nanotechnology (SAINT), Sungkyunkwan University, Suwon 16419, Korea; euyheon@skku.edu; 4 Department of Nano Science and Engineering, Kyungnam University, Changwon 51767, Korea; shbae@kyungnam.ac.kr; 5 Research Institute of Advanced Materials, Seoul National University, Seoul 08826, Korea

**Keywords:** thermoelectric, Tin Selenide, solid solution, Te substitution, spark plasma sintering

## Abstract

SnSe is considered as a promising thermoelectric (TE) material since the discovery of the record figure of merit (ZT) of 2.6 at 926 K in single crystal SnSe. It is, however, difficult to use single crystal SnSe for practical applications due to the poor mechanical properties and the difficulty and cost of fabricating a single crystal. It is highly desirable to improve the properties of polycrystalline SnSe whose TE properties are still not near to that of single crystal SnSe. In this study, in order to control the TE properties of polycrystalline SnSe, polycrystalline SnSe–SnTe solid solutions were fabricated, and the effect of the solid solution on the electrical transport and TE properties was investigated. The SnSe_1−x_Te_x_ samples were fabricated using mechanical alloying and spark plasma sintering. X-ray diffraction (XRD) analyses revealed that the solubility limit of Te in SnSe_1−x_Te_x_ is somewhere between x = 0.3 and 0.5. With increasing Te content, the electrical conductivity was increased due to the increase of carrier concentration, while the lattice thermal conductivity was suppressed by the increased amount of phonon scattering. The change of carrier concentration and electrical conductivity is explained using the measured band gap energy and the calculated band structure. The change of thermal conductivity is explained using the change of lattice thermal conductivity from the increased amount of phonon scattering at the point defect sites. A ZT of ~0.78 was obtained at 823 K from SnSe_0.7_Te_0.3_, which is an ~11% improvement compared to that of SnSe.

## 1. Introduction

Thermoelectric (TE) materials, which can reversibly convert thermal energy into electrical energy, have been considered as a way to solve the energy crisis and environmental problems [1,2]. The performance of a TE material is evaluated by the dimensionless figure of merit (ZT), ZT = (S^2^σ/k)T, where S, σ, k and T are the Seebeck coefficient, electrical conductivity, thermal conductivity, and absolute temperature, respectively [3]. Large ZT values can be obtained by high power factor (PF) (= S^2^σ) and low thermal conductivity. To increase both σ and S simultaneously is difficult because they tend to change in the opposite direction as the charge carrier concentration changes. It is also very difficult to increase σ and to decrease k at the same time as the electronic component of k tends to change in the same direction as the change of σ. So the effort to find TE materials which have a performance high enough to be used in devices, which consist of earth-abundant and non-toxic elements, is continuing today. 

Over the past several decades, various TE materials such as Bi_2_Te_3_-, PbTe-, and ZnSb-based compounds, skutterudites, and half-Heusler compounds have been integrated into TE devices [4,5,6,7]. In recent years, tin selenide (SnSe) which contains non-toxic and earth-abundant elements has been considered as a promising TE material since Zhao et al. [8] reported a remarkable ZT value of ~2.6 at 923 K (the world record to date) along the b-axis in its single crystal SnSe. It, however, is still difficult to use single crystal TE materials in devices because of poor mechanical properties and high production costs. For these reasons, the research on SnSe has focused on developing high performance polycrystalline SnSe [8,9,10,11]. Many approaches to obtain polycrystalline SnSe which has TE performance similar to that of single crystal through texturing [9], doping [10], and nanoinclusion [11] have been reported. The ZT values of polycrystalline SnSe which is lower than that of single crystal SnSe come mainly from its relatively low electrical conductivity (σ) and high thermal conductivity (k) [12]. Recently, Chung et al. [13] reported that the high thermal conductivity in polycrystalline SnSe is attributed to surface tin oxides and a ZT value of ~2.5 at 773 K in polycrystalline SnSe doped with 5% PbSe by removing surface oxide, which is the highest ZT value reported so far.

One potential way to optimize the TE performance of polycrystalline SnSe is by making a solid solution which has been used in other TE systems such as Bi_2−x_Sb_x_Te_3_, Mg_2_Si_1−x_Sn, and PbTe_1−x_Se_x_ [14,15,16,17]. In solid solution, electrical transport properties can be improved by optimizing the carrier concentration through band gap engineering while lattice thermal conductivity can be suppressed by enhanced phonon scattering through atomic disorder and mass difference [18]. To determine the element for forming the solid solutions, Hume-Rothery rules are usually used [19,20], which indicate that the solute and the solvent atoms should have: (1) similar size, (2) similar crystal structure, (3) similar valence state, and (4) similar electronegativity. 

There are a few reports on TE properties of SnSe-based solid solutions [21,22,23,24,25,26]. Han et al., [21] investigated the TE properties of SnS_1−x_Se_x_ solid solutions and reported that a ZT of ~0.82 in SnS_0.2_Se_0.8_, which was more than four times higher than that of SnS, was achieved by the increase of carrier mobility and the reduction of lattice thermal conductivity. Wei et al., [23] reported that single-phase Sn_1−x_Pb_x_Se solid solutions were formed up to x = 0.12, and the highest ZT was ~0.85 which was obtained in the sample with x = 0, which means that there was no significant enhancement in ZT value by Pb substitution. Saini et al., [24] reported that, the electrical properties of SnTe_x_Se_1−x_ (0 < x < 1) solid solutions which exhibit p-type conduction behaviours can be improved with the increase of Te contents. Hong et al., [25] also reported that SnSe_1−x_Te_x_ (0 < x < 0.2) nanoplates prepared with a solvothermal method have p-type conduction, and a ZT of ~1.1 was achieved in SnSe_0.9_Te_0.1_ which was higher than that of SnSe (ZT ~0.97). On the other hand, Chen et al., [26] reported that SnTe_x_Se_1−x_ (x= 0, 0.0625) has n-type conduction behaviour, and the incorporation of Te does not improve the electrical transport properties of SnSe. Thus, the precise role of Te on the TE properties of SnSe_1−x_Te_x_ solid solution is still not fully understood, and a detailed study of the TE transport properties of SnSe_1−x_Te_x_ solid solution is needed. 

The purpose of this study was to gain a better understanding of the effect of Te substitution on the TE properties of the SnSe–SnTe solid solution. In this study, SnSe_1−x_Te_x_ (0 ≤ x ≤ 1) were prepared by mechanical alloying and spark plasma sintering, and their TE transport properties were investigated. Here we report the solubility limit of Te in SnSe_1−x_Te_x_ is somewhere between x = 0.3 and 0.5 and a ZT of ~0.78 (x = 0.3) is obtained at 825 K (the ZT of SnSe is 0.7). The improvement of ZT is attributed to the increase of electrical conductivity which can be obtained from the tuning of carrier concentration and to the decrease of lattice thermal conductivity obtained by enhanced phonon scattering through point defect.

## 2. Materials and Methods 

Sn (99.99%, Sigma Aldrich, St. Louis, USA), Se (99.99%, Sigma Aldrich, St. Louis, USA) and Te (99.99%, Kunjundo Chemicals, Sakado, Japan) powders were used to synthesize the polycrystalline SnSe–SnTe solid solutions. High purity single elements of Sn, Se, and Te were weighed according to the stoichiometry of SnSe_1−x_Te_x_ (x = 0, 0.1, 0.3, 0.5, 0.8, and 1), loaded into a steel crucible (100 mL) at a ratio of 5:1 with steel balls of different diameters (3 mm and 1 mm) in an Ar filled glove box, and then subjected to mechanical alloying (MA). Approximately 35% of the crucible was filled with the powder mixture and balls. The polycrystalline SnSe_1−x_Te_x_ powders were synthesized by MA at 250 rpm for 4 h, which was confirmed by X-ray diffraction (XRD) (see the Figure S1 in supplementary material). In order to fabricate a bulk sample, the MA-derived powders were ground in an alumina mortar and sieved through a 140-mesh stainless steel mesh, loaded into a 12.5 mm diameter graphite mold and was spark plasma sintered in vacuum (~2 × 10^−3^ Torr) at 850 K for 10 min under uni-axial pressure of 30 MPa. 

The qualitative phase analyses of the disk-shaped sintered samples were carried out using X-ray diffraction (XRD, D-8 Advanced, Bruker, Karlsruhe, Germany) with Cu K_α_ radiation. The microstructures of fracture surface of the sintered samples were observed using a field emission scanning electron microscope (FE-SEM, SU-70, JEOL, Tokyo, Japan), and the elemental distribution analyses were conducted on the polished surface of bulk samples using an energy-dispersive X-ray spectroscopy (EDS, Oxford Instrument, Oxford, UK). The elemental ratios of the bulk samples were determined by electron probe micro-analysis (EPMA, JEOL, Tokyo, Japan). The dish-shaped sintered samples were cut into bars with dimensions of 1.5 × 1.5 × 7 mm^3^. The bar-shaped samples were used for simultaneous measurement of Seebeck coefficient and electrical conductivity using a commercial measurement equipment (Seepel Corp, Gunpo, Korea) under an Ar atmosphere from room temperature to 850 K. Carrier concentrations of the SnSe samples were obtained using a Hall measurement system (HMS 3000, Ecopia, Anyang, Korea) at room temperature. The optical characteristics of the SnSe_1−x_Te_x_ were determined using a UV-Vis-NIR spectrometer (UV-3600 plus, Shimadzu, Kyoto, Japan). The reflectance spectra were obtained over the wavelength range of 200 to 2600 nm with a step size of 1 nm. 

The analyses of density of state (DOS) and the calculations of electronic band structure were carried out for the SnSe_1−x_Te_x_ using the atomic positions and the lattice parameters (see the Table S1 in Supplementary Material) from the Rietveld analysis of each bulk sample. The unit cell of SnSe consists of eight atoms, and there are four sites of Se which can be substituted by Te per unit cell. Therefore, we used homogenous solid solutions of x = 0, 0.125 and 0.250 to calculate electronic band structures of SnSe_1−x_Te_x_.

We performed the first-principles calculation using the Vienna ab initio simulation package (VASP) based on the density functional theory (DFT) [27]. The exchange and correlation energies were treated within the generalized gradient approximation (GGA) according to the Perdew–Burke–Ernzerhof (PBE) parameterization. Projector augmented-wave (PAW) potentials are used for electron-ion interactions. A plane wave kinetic energy cutoff of 450 eV and 4 × 12 × 12 k-point sets were used. The self-consistent calculation for structural optimization cycles were repeated until the energy difference and force became smaller than 1 × 10^−6^ eV and 1 × 10^−2^ e V Å^−1^, respectively. The standard PBE+U calculations were used to obtain the band gap energy. 

The total thermal conductivity (k_tot_) can be expressed as the sum of electronic thermal conductivity (k_e_) and lattice thermal conductivity (k_latt_). The k_tot_ was calculated by multiplying the specific heat (C_p_), thermal diffusivity (D), and density (ρ). The C_p_, D, and ρ values were measured using the differential scanning calorimetry (DSC, Netzsch, Selb, Germany), the laser flash method (LFA457, Netzsch), and the Archimedes method, respectively. The k_e_ was obtained using the Wiedemann–Franz law, ke = LσT, where L, σ and T are Lorenz number, electrical conductivity, and absolute temperature, respectively. The Lorenz number L which was obtained from fitting the Seebeck coefficient to the reduced chemical potential was used to obtain k_e_ [28,29]. The k_latt_ was obtained by subtracting the k_e_ from the k_tot_. The TE properties of all samples were measured perpendicular to the direction of the pressure applied during the SPS. 

## 3. Results and Discussion

**Figure 1:**
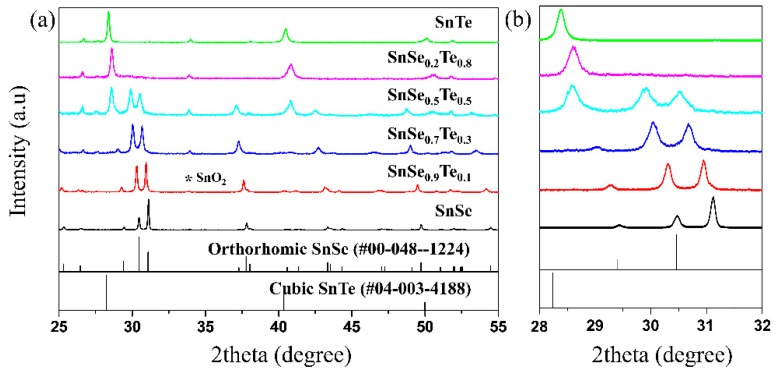
(**a**) Theta-2theta X-ray diffraction (XRD) patterns of the polycrystalline SnSe_1−x_Te_x_ (x = 0, 0.1, 0.3, 0.5, 0.8 and 1) and (**b**) featured 2θ profiles on expanded scale.

Figure 1 shows the XRD patterns of the sintered SnSe_1−x_Te_x_ (x = 0, 0.1, 0.3, 0.5, 0.8, and 1) prepared using MA and spark plasma sintering. For all the samples, there is a peak at around 2 theta = 34 which corresponds to SnO_2_, which is consistent with the results of previous reports [30,31]. Zhang et al. [31] reported that oxygen can be physically adsorbed on SnSe before SPS, and it can be chemically adsorbed on SnSe after SPS and transformed into oxides. However, the Sn-rich phase is not observed by EDS, and chemical compositions of all samples are close to the nominal ones (shown in Figure 3 and Table 1). Therefore, we assumed that the amount of SnO_2_ inside the sintered samples is very small and almost the same in all the sintered samples, so the effect of SnO_2_ on the TE properties was not accounted for in this study. When x = 0, all the peaks of the sample are matched with those of orthorhombic SnSe (PDF #00–048–1224). On the other hand, when x = 1, all the peaks of the sample are matched with those of cubic SnTe (PDF #04–003–4188). As Te contents are increased, the peak positions of the samples tend to shift towards low 2θ values, which indicates that the incorporation of Te induces lattice expansion (see Table S1 in Supplementary Material) due the fact that Te has a larger atomic radius (0.21 nm) than Se (0.19 nm), which is consistent with the results previous reported [25]. When x = 0.5, diffraction peaks from SnSe and SnTe are observed simultaneously, which indicates the presence of both SnSe and SnTe. This implies that the solubility limit of Te element in SnSe_1−x_Te_x_ can be somewhere between x = 0.3 and 0.5. In this study, all the SnSe_1−x_Te_x_ samples were sintered at 850 K. Volykhov et al. [32] reported that the solubility limit of Te in SnSe which depends on the temperature is ~0.35 at ~850 K, which is consistent with the result of this study. The microstructures and TE properties of three samples (SnSe, SnSe_0.9_Te_0.1_, and SnSe_0.7_Te_0.3_) with Te content smaller than the solubility limit, were observed and measured, respectively, in this study.

**Figure 2:**
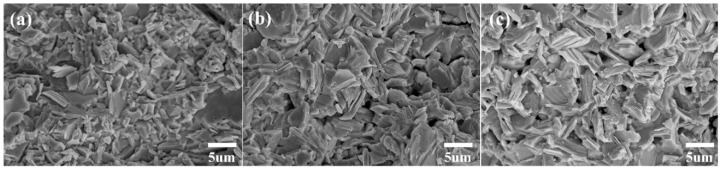
Field emission scanning electron microscope (FE-SEM) micrographs taken from the fracture surface of the polycrystalline (**a**) SnSe, (**b**) SnSe_0.9_Te_0.1_, and (**c**) SnSe_0.7_Te_0.3_. The surfaces of the samples were not polished.

**Figure 3:**
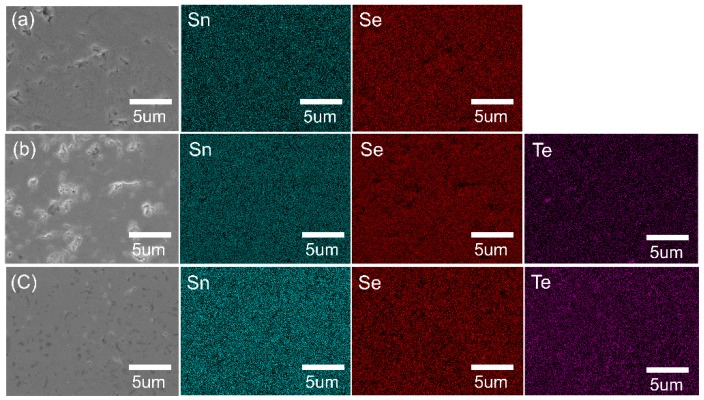
Electron probe microanalyzer (EPMA) images of (**a**) SnSe, (**b**) SnSe_0.9_Te_0.1_, and (**c**) SnSe_0.7_Te_0.3_. Images with (Sn), (Se), and (Te) show the distribution of the elements Sn, Se, and Te, respectively.

Figure 2a–c show the FE-SEM micrographs taken from the fracture surface of the polycrystalline SnSe_1−x_Te_x_ (x = 0, 0.1 and 0.3) samples, respectively. All the sintered SnSe_1−x_Te_x_ samples exhibit dense microstructure and plate-like grains. As the amount of Te is increased, no significant microstructural changes are observed. The relative densities of all samples measured by the Archimedes method were ~95%, and it can be expected that the effect of density on the TE properties of the SnSe_1−x_Te_x_ samples can be negligible. In order to determine the distribution of elements, EDS mapping was performed on the polished surface of the polycrystalline SnSe_1−x_Te_x_ (x = 0, 0.1, and 0.3), and the results are shown in Figure 3. EDS elemental mapping results show that the distributions of the element Sn, Se, and Te are uniform. The atomic ratios and chemical compositions of all samples were determined by electron probe microanalysis (EPMA), and the result are shown in Table 1. The Sn/Se/Te atomic ratios of SnSe, SnSe_0.9_Te_0.1_, and SnSe_0.7_Te_0.3_ are 49.9/50.1/0, 50.1/45.2/4.7, and 50.2/35.3/14.5, respectively, which indicates that the analyzed chemical compositions of all samples are close to the nominal ones. 

**Figure 4:**
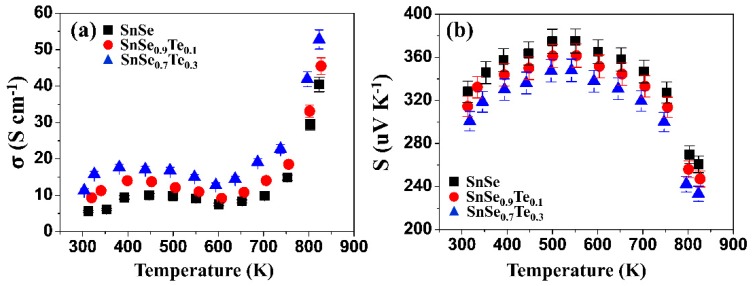
The temperature-dependency of (**a**) electrical conductivity (σ) and (**b**) Seebeck coefficient (S) of the polycrystalline SnSe_1−x_Te_x_ (x = 0, 0.1, and 0.3).

Figure 4a shows the temperature-dependency of electrical conductivity (*σ*) of the polycrystalline SnSe_1−x_Te_x_ (x = 0, 0.1, and 0.3). All the curves have the same trend of change: σ is increased with temperature first, decreased from 473 K to 700 K, and then rapidly increased over 790 K again thereafter, which is consistent with the results of previous reports [9,33,34,35]. Sassi et al. [33] and Zhang et al. [34] reported a reduction of σ around 505 K due to the melting of a very small amount of unreacted Sn. Feng et al. [35] and Fu et al. [9] reported that an increase of σ at about 623 K can be attributed to the thermal activation of minority carriers and a rapid increase of σ at 750–800 K is due to the phase transition of SnSe from Pnma to Cmcm. Zhao et al. [30] reported an anomalous jump in the heat capacity come from the phase transition. The heat capacity of SnSe measured by differential scanning calorimetry (DSC) (see Figure S2 in supplementary material) showed that the phase transition may occur at around 790 K. As the amount of Te is increased, the σ values are increased, and SnSe_0.7_Te_0.3_ has higher electrical conductivity than the other two at all the measurement temperatures. To understand the variations in electrical conductivity with increasing Te contents, the carrier concentration (*n*) and carrier mobility (*μ*) of all samples were measured at room temperature, and the results are shown in Table 2. The *n* values of all the samples are positive, which indicates that hole is the major carrier and SnSe_1−x_Te_x_ is a p-type semiconductor. As the Te contents are increased, *n* is increased from 3.2 × 10^18^ to 1.3 × 10^19^, while *μ* is decreased from 10.5 to 5.1 cm^−2^ V^−1^ s^−1^, which means that the improvement in the electrical conductivity can be attributed to the increase of carrier concentration. 

Figure 4b shows the temperature-dependency of Seebeck coefficients (S) of the polycrystalline SnSe_1−x_Te_x_ (x = 0, 0.1, and 0.3). The change of S values with the increase of temperature in all samples shows the same behavior: S was increased from room temperature to 550 K, and then decreased as temperature is further increased. The slight decrease in S value in the temperature range of 550–673 K is known to result from the bipolar conduction by the excitation of the minor carrier [35]. The sharp decrease in S values from 790 K is known as the result of the increase of carrier concentration by the phase transition of SnSe from Pnma to Cmcm [36]. Unlike the electrical conductivity, the S values of the samples are decreased with the increase of amount of Te. Generally, S values are determined by the Pisarenko relationship [37], which indicates that the S value is inversely proportional to the carrier concentration. As shown in Table 2, the carrier concentration was increased with the increase of Te content, which can lead to the decrease of the Seebeck coefficient. Therefore, the change in the electrical conductivity and the Seebeck coefficient can be influenced by the increase in carrier concentration. The carrier concentration is closely related to the band gap energy, and its relation is given below [38].

(1) $$n_{i}=CT^{3/2}\exp\left(\frac{-E_{g}}{2K_{B}T}\right)$$

where *n_i_*, *C*, *T*, *k_B_*, and *E_g_* are the carrier concentration, a constant, the absolute temperature, the Boltzmann constant, and the band gap energy, respectively. The carrier concentration of a material is inversely proportional to the band gap energy, which means that a reduction of the band gap can lead to the increase in carrier concentration. To investigate the band gaps of SnSe_1−x_Te_x_ (x = 0, 0.1, and 0.3) solid solutions, the band gap of each sample was determined from its absorption coefficient (α) obtained by UV-Vis-NIR spectroscopy. The relation between band gap energy (E_g_) and the absorption coefficient (α) for indirect band transition can be established by the Tauc relation given below [39].

(*αhν*)^1/2^ = *A*(*hν* − *E_g_*) (2)

where *A*, *h*, and *ν* are a constant, the Planck’s constant, and the frequency of radiation, respectively.

**Figure 5:**
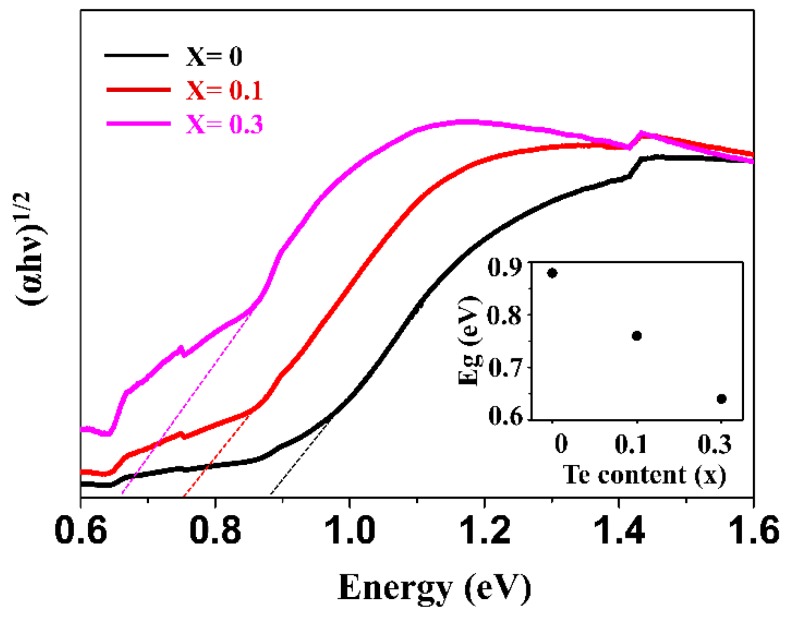
(*αhv*)^1/2^ versus (*hν*) plots of the polycrystalline SnSe_1−x_Te_x_ (x = 0, 0.1 and 0.3) obtained by UV-Vis-NIR spectrum. The inset shows the optical band gap versus Te content. The band gap value for each sample was obtained by extrapolating these plots to the x-axis.

Figure 5 shows (*αhv*)^2^ vs. (*hν*) plots of the polycrystalline SnSe_1−x_Te_x_ (x = 0, 0.1 and 0.3). The optical band gap of each sample was obtained from the extrapolation of the linear region of the plot to the *hν* axis, and the results are shown in the inset of Figure 5. The obtained band gap of SnSe was 0.88 eV, and it is in good agreement with the result of optical absorption measurement (0.90 eV) reported by Shi et al [40]. The band gaps of SnSe_0.9_Te_0.1_ and SnSe_0.7_Te_0.3_ were 0.76 and 0.64 eV, respectively, which indicates that the band gaps of SnSe_1−x_Te_x_ are decreased with increasing Te contents, which is consistent with the results reported by Wei et al [21]. To further understand the decrease in band gap, density of states (DOS) and band structures of SnSe_1−x_Te_x_ were obtained using DFT calculations. The bandgaps measured using UV-VIS-NIR spectrum and those calculated by DFT were compared.

**Figure 6:**
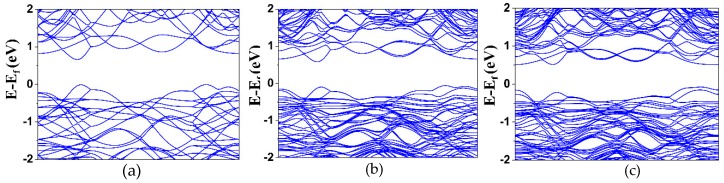
Electronic band structures of the polycrystalline (**a**) SnSe, (**b**) SnSe_0.725_Te_0.125_, and (**c**) SnSe_0.75_Te_0.25_, respectively.

Figure 6a–c show the electronic band structures of the polycrystalline SnSe_1−x_Te_x_ (x = 0, 0.125, and 0.25), respectively. The band structures were obtained by the first principle calculations using the VASP based on the DFT. The first and the second conduction band minima (CBM1 and CBM2) are shown in the Γ-F and Z-Γ directions, respectively, and the first and second valence band maxima (VBM1 and VBM2) are shown in the Z-Γ and Γ-F directions, respectively, which means that the SnSe_1-x_Te_x_ have indirect band gap. The calculated band gap of SnSe was 0.68 eV, and it is different from the measured band gap of SnSe, which was 0.88 eV (Figure 5). Chen et al. [26] and Su et al. [41] reported that the difference between measured and calculated bandgap can be caused by a well-known drawback from the standard DFT calculation. The measurement and the calculation of the band gap energy show widen band gaps of the SnSe_1−x_Te_x_ with the increase of Te content. 

**Figure 7:**
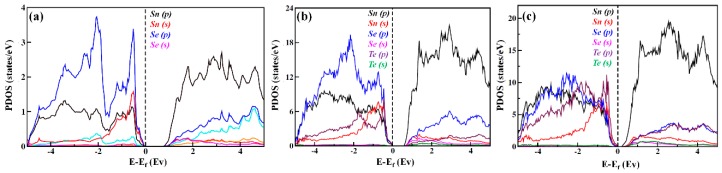
The projected density of states per atom of (**a**) SnSe, (**b**) SnSe_0.725_Te_0.125_, and (**c**) SnSe_0.75_Te_0.25_, respectively.

Figure 7a–c shows the projected density of states of SnSe_1−x_Te_x_. For SnSe, the valence band maxima (VBM) is mainly contributed by Se-p orbital, while the conduction band minima (CBM) is occupied by Sn-p orbital. With increasing Te contents, VBM is mainly contributed by Sn-s and Te-p orbital hybridization in the VB, while the major part of the CBM is occupied by Sn-p orbital. The Sn-p orbital was shifted toward the Fermi level (E_F_), leading to the CBM down to the Fermi level (E_F_). This resulted in a band gap reduction, which is consistent with the result reported by Chen et al [26]. They reported that the interaction between Sn and Te orbitals can move the CBM towards the Fermi level (E_F_) and consequently reduce the band gap. Therefore, the increase of electrical conductivity and the decrease of the Seebeck coefficient in this study are mainly caused by the increase of carrier concentration due to the band gap reduction.

**Figure 8:**
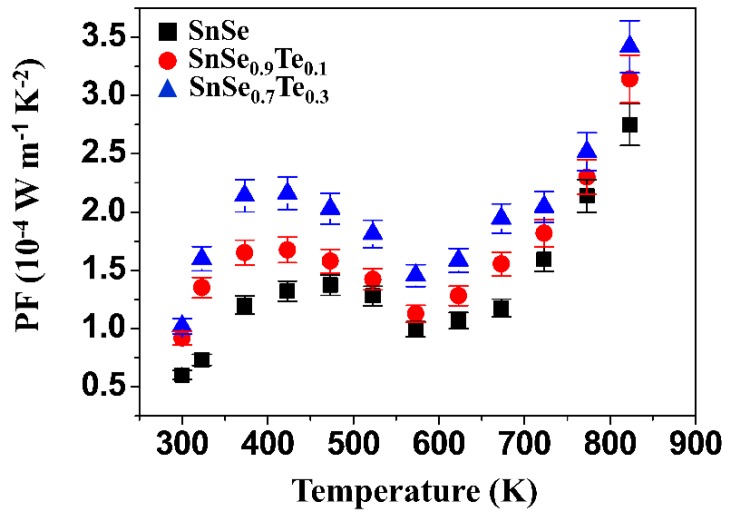
The power factor (PF) of the polycrystalline SnSe_1−x_Te_x_ (x = 0, 0.1 and 0.3) obtained at different temperatures.

Figure 8 shows the power factor (PF = S^2^σ) of the polycrystalline SnSe_1–x_Te_x_ obtained at different temperatures. The SnSe_0.7_Te_0.3_ sample shows a relatively high PF compared to the SnSe at all measurement temperatures, and the PF of SnSe_0.7_Te_0.3_ was ~1.02 × 10^−4^ W m^−1^ K^−1^ at 300K and ~3.42 × 10^−4^ W m^−1^ K^−1^ at 823 K (this was the highest PF obtained in this study), which can mainly come from the improved electrical conductivity.

**Figure 9:**
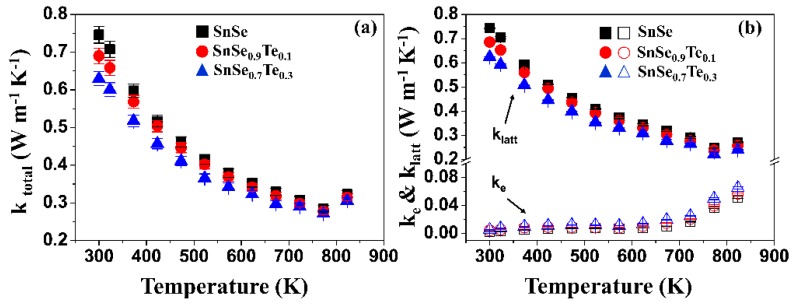
The temperature-dependency of (**a**) total thermal conductivity (k_tot_), (**b**) electronic thermal conductivity (k_e_), and the lattice thermal conductivity (k_latt_) of the polycrystalline SnSe_1−x_Te_x_. (hollow shapes - k_e_, filled shapes - k_latt_).

Figure 9a shows the temperature-dependency of total thermal conductivities (k_tot_) of the polycrystalline SnSe_1−x_Te_x_. All samples show the same behavior with temperature: k_tot_ is generally decreased up to 790 K, and then slightly increased. The increase in k_tot_ is known as the result of the phase transition of the SnSe from Pnma to Cmcm [42]. k_tot_ of SnSe_0.7_Te_0.3_ is lower than that of SnSe at all measurement temperatures. k_tot_ is expressed as the sum of electronic thermal conductivity (k_e_) and lattice thermal conductivity (k_latt_). In order to better understand the low k_tot_ of SnSe_0.3_Te_0.7_, the k_tot_ of all samples were separated into k_e_ and k_latt_, and the results are shown in Figure 9b. The k_tot_ and k_latt_ of SnSe at 300 K are 0.75 and 0.74 W m^−1^ K^−1^, respectively, which indicates that the k_tot_ comes mainly from the k_latt_. The k_latt_ of the samples were decreased with the increase of Te content. The atomic masses of Se and Te are 78.96 g mol^−1^ and 127.6 g mol^−1^, and the atomic radius of them are 0.19 nm and 0.21 nm, respectively. The atomic mass and size difference between Se and Te can cause the fluctuation of mass and strain field, which can lead to the increase in the phonon scattering at point defects [21]. Therefore, the decrease in k_latt_ with increasing Te content can come from the increased amount of phonon scattering. The k_latt_ can be explained in terms of Umklapp scattering which is used for defect-free crystalline materials [43]. The polycrystalline SnSe, however, can have a lot of defects due to Te substitution, and so the phonon scattering by point defects is used to explain the decrease in k_latt_ with the increase of Te content in this study. 

**Figure 10:**
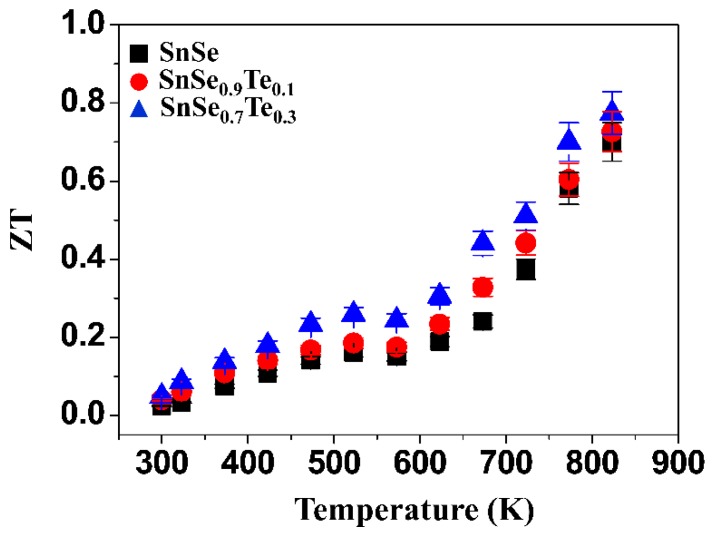
The figure of merits (ZTs) of the polycrystalline SnSe_1−x_Te_x_ measured at different temperatures.

Figure 10 shows the figure of merits (ZTs) of the polycrystalline SnSe_1−x_Te_x_, which were obtained from the electrical conductivity, Seebeck coefficient and thermal conductivity measured at different temperatures. The ZT value of SnSe_0.7_Te_0.3_ is higher than that of SnSe at all measurement temperatures. The highest ZT obtained in this study was ~0.78 at 823 K of SnSe_0.7_Te_0.3_, which can be attributed to the improvement of electrical conductivity and the reduction of lattice thermal conductivity. 

## 4. Conclusions

In this study, the thermoelectric (TE) properties of polycrystalline SnSe_1−x_Te_x_ solid solutions were investigated. SnSe–SnTe solid solutions were prepared by mechanical alloying and spark plasma sintering. XRD and EPMA analyses showed that the solubility limit of Te in SnSe_1−x_Te_x_ is somewhere between x = 0.3 and 0.5. Hall measurement showed that carrier concetration was increased with increasing Te contents. The measurements of band gap using UV-VIS-NIR spectrum and calculation of band gap from DFT showed that the band gap was decreased as the amount of Te was increased, which can lead to the increase of carrier concentration. The increase in electrical conductivity and the reduction of Seebeck coefficeint of SnSe_1−x_Te_x_ were observed, which can result from the increase of carrier concentration. The thermal conductivity was decreased with increasing Te contents. Te has a larger atomic mass and size than Se, and the presence of Te at Se site can act as an effective point defect, which can increase phonon scattering and reduce lattice thermal conductivity. A ZT of ~0.78 was obtained at 823 K from the SnSe_0.7_Te_0.3_, which was ~11% higher than that of SnSe. This study shows that polycrystalline SnSe_1−x_Te_x_ can have improved TE properties compared to SnSe which can be attributed to the increase in electrical conductivity and the decrease in the lattice thermal conductivity.

## Figures and Tables

**Table 1:** Tin (Sn), selenium (Se), and tellurium (Te) atomic ratios and chemical compositions of polycrystalline SnSe_1−x_Te_x_ (x = 0, 0.1 and 0.3) obtained by electron probe micro-analyses (EPMA).

Nominal Compositions	Sn (at %)	Se (at %)	Te (at %)	Analyzed Compositions
SnSe	49.9	50.1	–	Sn_0.998_Se_1.002_
SnSe_0.9_Te_0.1_	50.1	45.2	4.7	Sn_1.002_Se_0.904_Te_0.094_
SnSe_0.7_Te_0.3_	50.2	35.3	14.5	Sn_1.004_Se_0.706_Te_0.290_

**Table 2:** Charge transport properties of polycrystalline SnSe_1−x_Te_x_ (x = 0, 0.1, and 0.3). The carrier concentration (n), carrier mobility (μ), and electrical conductivity (σ) were measured at room temperature.

Samples	Hall Carrier Concentration (n, cm^−3^)	Carrier Mobility (μ, cm^−2^ V^−1^ s^−1^)	Conductivity (σ, S cm^−1^)
SnSe	3.16 × 10^18^	10.46	5.59
SnSe_0.9_Te_0.1_	8.38 × 10^18^	6.56	9.30
SnSe_0.7_Te_0.3_	1.31 × 10^19^	5.09	11.29

## Supplementary Materials

The following are available online at https://www.mdpi.com/1996-1944/12/23/3854/s1, Figure S1: Theta-2theta XRD patterns of the polycrystalline SnSe_1−x_Te_x_ (x = 0, 0.1, 0.3, 0.5, 0.8 and 1) powder prepared using mechanical alloying, Figure S2: Heat capacity of SnSe measured by differential scanning calorimetry (DSC), Table S1: The cell parameters of the polycrystalline SnSe_1−x_Te_x_ (x = 0, 0.1, 0.3, 0.5, 0.8 and 1) obtained by Rietveld refinement method using TOPAS software.
