# Supplementary material for: Electrical Transport and Thermoelectric Properties of SnSe–SnTe Solid Solution
Source: Materials (Basel). 2019 Nov 22;12(23):3854. doi: 10.3390/ma12233854 (PMC6926679; doi:10.3390/ma12233854)
Supplement: Supplementary file 1 [file materials-12-03854-s001.pdf]

materials

MDPI

Article

Electrical Transport and Thermoelectric Properties of SnSe–SnTe Solid Solution

Jun-Young Cho [1], Muhammad Siyar [2], Woo Chan Jin [1], Euyheon Hwang [3], Seung-Hwan Bae [4], Seong-Hyeon Hong [1], Miyoung Kim [1] and Chan Park [1,5,*]

[1] Department of Materials Science and Engineering, Seoul National University, Seoul 08826, Republic of Korea; takecjy@gmail.com (J.-Y.C.); woottan@snu.ac.kr (W.C.J.); shhong@snu.ac.kr (S.-H.H.); mkim@snu.ac.kr(M.K.)

[2] School of Chemical & Materials Engineering, National University of Sciences and Technology, Islamabad H–12, Pakistan; engrsiyar.uet@gmail.com

[3] SKKU Advanced Institute of Nanotechnology (SAINT), Sungkyunkwan University, Suwon 16419, Republic of Korea; euyheon@skku.edu

[4] Department of Nano Science and Engineering, Kyungnam University, Changwon 51767, Republic of Korea; shbae@kyungnam.ac.kr

[5] Research Institute of Advanced Materials, Seoul National University, Seoul 08826, Republic of Korea

* Correspondence: pchan@snu.ac.kr

Received: 28 October 2019; Accepted: 19 November 2019; Published: date

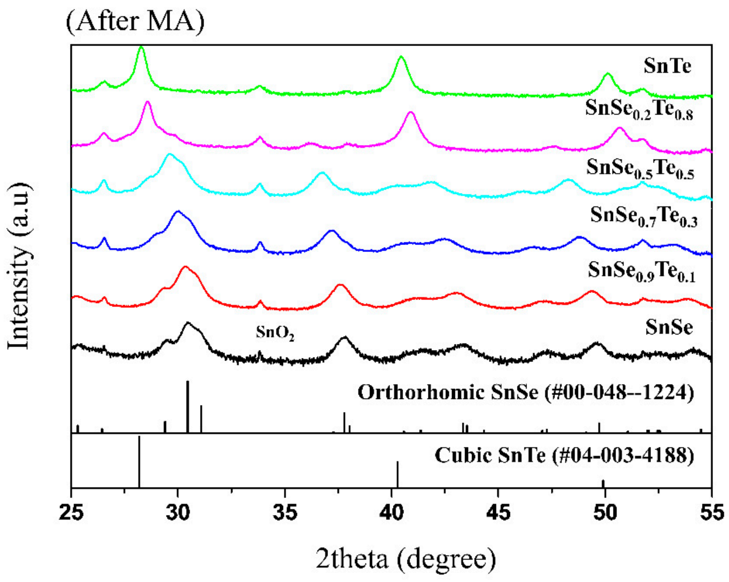

Figure S1. Theta-2theta XRD patterns of the polycrystalline $SnSe_{1-x}Te_x$ (x= 0, 0.1, 0.3, 0.5, 0.8 and 1) powder prepared using mechanical alloying.

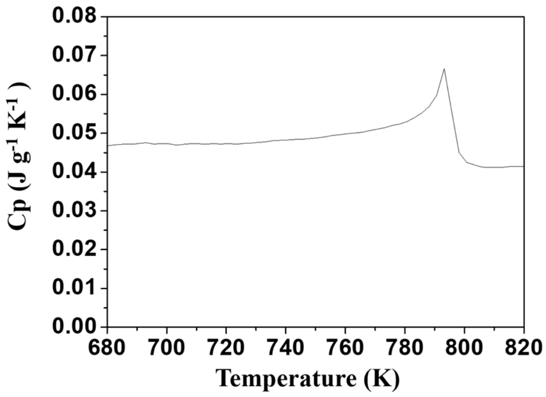

Figure S2. Heat capacity of SnSe measured by differential scanning calorimetry (DSC).

Table S1. The cell parameters of the polycrystalline $SnSe_{1-x}Te_x$ (x = 0, 0.1, 0.3, 0.5, 0.8 and 1) obtained by Rietveld refinement method using TOPAS software.

Sample	Composition	a(Å)	b(Å)	c(Å)	$\alpha=\beta=\gamma$
1	SnSe	11.495	4.152	4.442	90
2	$Sn(Se_{0.9}Te_{0.1})$	11.542	4.183	4.458	90
3	$Sn(Se_{0.7}Te_{0.3})$	11.659	4.227	4.483	90
4	$Sn(Se_{0.5}Te_{0.5})$	11.703	4.251	4.489	90
		6.243	6.243	6.243	90
5	$Sn(Se_{0.2}Te_{0.8})$	6.261	6.261	6.261	90
6	SnTe	6.315	6.315	6.315	90
